# Expression and Methylation of Mitochondrial Transcription Factor A in Chronic Obstructive Pulmonary Disease Patients with Lung Cancer

**DOI:** 10.1371/journal.pone.0082739

**Published:** 2013-12-18

**Authors:** Hong Peng, Min Yang, Zhi-yong Chen, Ping Chen, Cha-xiang Guan, Xu-dong Xiang, Shan Cai, Yan Chen, Xiang Fang

**Affiliations:** 1 Department of Respiratory Medicine, The Second Xiangya Hospital of Central-South University, Changsha, Hunan, PR China; 2 Human Key Laboratory of Medical Epigenomics, The Second Xiangya Hospital of Central-South University, Changsha, Hunan, PR China; ? Department of Urology, Xiangya Hospital of Central-South University, Changsha, Hunan, PR China; 4 Physiological Research Center, Xiangya Medical School of Central-South University, Changsha, Hunan, PR China; 5 Department of Neurology, University of Texas Medical Branch, Galveston, Texas, United States of America; CEA - Institut de Genomique, France

## Abstract

**Background:**

Apoptosis plays a central role in the pathogenesis of chronic obstructive pulmonary disease (COPD), and this process can be regulated by mitochondrial transcription factor A (mtTFA). Epigenetics is involved in the regulation and modification of the genes involved in lung cancer and COPD. In this study, we determined the expression of mtTFA and its methylation levels in the COPD patients with lung cancer.

**Methods:**

Twenty-one squamous cell lung cancer patients, 11 with COPD and 10 without COPD, undergoing pneumonectomy were enrolled. The apoptotic index (AI) of pulmonary vascular endothelial cells was analyzed by transferase-mediated deoxyuridine triphosphate-biotin nick end labeling assay. The expression of mtTFA mRNA and protein was measured using PCR, immunohistochemistry and Western-blot. Methylation of the *mtTFA* promoter was detected using bisulfite sequencing PCR.

**Results:**

Compared to the non-COPD group, the AI was higher, and expression of mtTFA mRNA and protein was lower in the COPD group (*P*<0.001). Expression of the mtTFA protein was positively correlated with FEV_1_/Pre (*r* = 0.892, *P*<0.001), and negatively correlated with AI (*r* = −0.749, *P*<0.001) and smoke index (*r* = −0.763, *P*<0.001). Percentage of *mtTFA* promoter methylation in the COPD patients was significantly higher compared to the non-COPD patients (*P*<0.05).

**Conclusion:**

These results suggest that the expression of mtTFA mRNA and protein is down-regulated in the lung tissue from the COPD patients with squamous cell lung cancer, and the level of mtTFA protein is related to apoptosis of pulmonary vascular endothelial cells. Aberrant *mtTFA* methylation may also play an important role in the pathogenesis of COPD.

## Introduction

Chronic obstructive pulmonary disease (COPD) is one of the most common chronic diseases. The global prevalence in adults over 40 years of age is estimated to be 10% [1]. Many mechanisms such as chronic inflammation, proteinase/anti-proteinase imbalance, and oxidative stress are involved in the pathogenesis of COPD [2], and they interact with each other during the development of the disease. Recent data from both animal models and human studies [3]–[6] suggests an important role for endothelial apoptosis in the pathological processes of COPD.

Mitochondrial transcription factor A (mtTFA, TFAM) is a nucleus-encoded protein that binds upstream of the light strand promoter (LSP) and heavy strand promoter (HSP) of mitochondrial DNA (mtDNA). mtTFA promotes transcription of mtDNA and regulates mtDNA replication [7]. Disruption of the *mtTFA* gene in mice results in depletion of mtDNA, loss of mitochondrial transcripts, impairment of chain respiration, cell apoptosis, and reduction of mtDNA-encoded polypeptides [8], [9]. On the other hand, overexpression of mtTFA can ameliorate the decline in mtDNA copy number and apoptosis in transgenic (Tg) mice [10]. Remels et al [11] found that the amount of mtTFA protein was significantly lower in the quadriceps muscle of patients with moderate-to-very severe COPD than in controls. They also reported that amount of mtTFA mRNA and protein were significantly lower in patients with cachectic COPD compared to that in both non-cachectic patients and control subjects [11]. These observations suggest that the disruption of mtTFA is associated with skeletal muscle abnormalities in patients with COPD. However, the expression of mtTFA in the lung of COPD patients has not been studied and the underlying mechanisms have not been elucidated.

Epigenetics refers to heritable changes in gene function that occur through changes in chromatin structure without any change in the DNA sequence. One of the key epigenetic mechanisms is DNA methylation, which represses gene transcription, and modifies the core histones of impaired DNA in the chromosome. DNA methylation can result in either activation or repression of genes [12]. Up to date, most of the research in this area is focused on cancer, cellular development and differentiation. However, there is increasing evidence that epigenetics may also play an important role in the regulation of genes involved in asthma and COPD [13]. Demeo et al found that variable DNA methylation is associated with COPD and lung function [14]. They also found that *Alu* and LINE-1 hypomethylation are associated with lower lung function and/or rapid lung function decline in the elderly patients [15]. Therefore, epigenetic aetiology presents a novel target for the treatment of asthma and COPD [16].

Cigarette smoke is a key risk factor for COPD. Numerous studies have shown that smoking can alter DNA methylation. One previous report showed that there was at least one aberrant gene methylation in 32 percent of bronchial brush specimen from persons with a smoking history [17]. Kikuchi et al [18] also found TSLC1/IGSF4 methylation was correlated with smoking index. Cigarette smoking and time interval of abstinence are associated with differential DNA methylation across the human genome [19]. Sequence analysis revealed that there are 67 potential CpG methylation loci between −2246 bp and +132 bp of the human *mtTFA* promoter, suggesting that cytosine methylation may play a role in the regulation of mtTFA expression. Choi et al [20] found the luciferase activities of pGL2-hTfam in transiently transfected HepG2 cells is suppressed by site-specific methylation. This *in vitro* study demonstrated that methylation of nuclear respiratory factor 1 (NRF-1) binding region strongly inhibited the activity of the *mtTFA* promoter in transiently transfected HepG2 cells.

In the present study, we tested the hypothesis that changes in expression of mtTFA and aberrant *mtTFA* methylation might play important roles in the pathogenesis of COPD.

## Materials and Methods

### Ethic Statement

This study was approved by the institutional ethics committee of the Second Xiangya Hospital of Central-South University and written informed consent was obtained from all subjects before their enrollment into the study.

### Patients and their general information

The study population consisted of 11 squamous cell lung cancer patients (stage II) with COPD and 10 squamous cell lung cancer patients (stage II) without COPD who underwent pneumonectomy. The lung tissue 5 cm away from tumor margin was used in our studies. All COPD patients suffered from chronic airflow limitation, defined as a post-bronchodilator forced expiratory volume in one second over forced vital capacity (FEV_1_/FVC) is <70%, in the absence of alternative causes. All the patients had not received lung transplant, radiotherapy, chemotherapy or inhaled corticosteroid therapy. The enrolled subjects did not have any other confoundable medical conditions such as gastrointestinal, renal, endocrine, metabolic and inflammatory diseases. FEV_1_ and FVC were assessed from the flow-volume curve obtained using a spirometer (Sensormedics AutoBox-6200D, USA). Lung function parameters were expressed as a percentage of reference values. General patient characteristics are shown in table 1. There were no differences in age or gender between the two groups (*P*>0.05). Compared to the non-COPD group, the smoking index of the COPD group was significantly higher (*P*<0.001), while both FEV_1_/Pre (%) (percentage of FEV_1_ actual of FEV_1_ predicated) and FEV_1_/FVC (%) were lower in the COPD group (*P*<0.001).

**Table 1:** The general state of the two groups of patients.

		Non-COPD group	COPD group	P value
sex	male	7	9	
	female	3	2	
age(y)		52.5±12.1	59.6±14.7	0.09
smoking index(pack year)		12.1±8.9	33.6±12.0***	0.0002
FEV_1_/Pre (%)		95.4±6.1	70.5±9.0***	0.0000008
FEV_1_/FVC (%)		79.7±6.1	60.8±3.3***	0.0000006

*P*<0.001 compare with non-COPD group.

### Methods

#### Histological studies

Paraffin-embedded sections of human lung tissue were deparaffinized and rehydrated with xylene and ethanol. The sections were briefly washed, stained in Harris hematoxylin solution for 5 to 10 minutes, differentiated in 1% acid alcohol for 30 seconds, and then washed. The sections were then counterstained in eosin-phloxine solution for 30 seconds to 1 minute, dehydrated with 95% alcohol and 2 changes of absolute alcohol for 5 minutes each, cleared in 2 changes of xylene 5 minutes each, and mounted with xylene based mounting medium. Histology was assessed by a pathologist in a blinded fashion. As emphysema is a structural disorder characterized by destruction of the alveolar walls and enlargement of the alveolar spaces, enlargement of alveolar spaces was assessed by quantifying the mean linear intercept (MLI) and destruction of alveolar walls by measuring the destructive index (DI). The MLI, a measure of inter-alveolar wall distance, was determined by light microscopy at a total magnification of 100×. Fields that included large airways or vessels were excluded from the analysis. The MLI was defined as the total length of the cross-line divided by the numbers of alveolar walls intersecting the test lines, as described by Xu et al [21]. The DI was calculated as a measure of parenchymal destruction using a microscopic point-count technique [22]. The analysis was performed in duplicate by randomly counting more than 3,000 alveoli from 50 HE sections from each patient at a magnification of 200×.

#### Measurement of apoptosis

Terminal Deoxynucleotidyltransferase-Mediated dUTP Nick End Labeling (TUNEL) Assay was used to measure the apoptosis of endothelial cells in the lungs of COPD patients. Cryostat tissue sections of lung were fixed in 1% paraformaldehyde in PBS for 10 min at room temperature. TUNEL staining was carried out with the *In Situ* Cell Death Detection Kit-POD (ROCHE). TUNEL positive and negative cells on the whole section were counted by a pathologist in a blinded fashion, and the apoptotic index was calculated as the number of TUNEL-positive endothelial cells/whole endothelial cells.

#### Reverse Transcriptase-Polymerase Chain Reaction (RT-PCR)

Total RNA was extracted using Trizol Reagent one-step extraction method (Invitrogen, USA). RNA concentration was determined using a spectrophotometer and the quality was verified by agarose gel electrophoresis. RNA (2 mg per sample) was reverse transcribed to make complementary DNA (cDNA) according to the manufacturer's instructions (RT kit,FERMENTAS,USA). cDNA was PCR amplified using Platinum Taq DNA polymerase (Invitrogen, Breda, the Netherlands) on a thermal cycler apparatus (Applied B PE4500, USA). Thermal cycling conditions were designed as follows: initial denaturation at 95°C for 5 min, followed by 35 cycles at 94°C for 30 sec and 55°C for 45 sec, and an extension step of 5 min at 72°C. The primer pairs used for PCR were as follows: 5′-TATCAAGATGCTTATAGGGC-3′ and 5′-ACTCCTCAGCACCATATTTT-3′ for mtTFA (amplicon expected size: 441 bp); 5′-ACCCACACTGTGCCCATCTAC-3′ and 5′-TCGGTGAGGATCTTCATGAGGTA-3′ for β-actin (amplicon expected size: 103 bp). Transcript level of the constitutive housekeeping gene β-actin was quantitatively measured as a control of RNA loading. Gene expression was quantified and expressed in arbitrary units (AU).

#### Real-time RT-PCR (qRT-PCR)

Isolated RNA was reverse transcribed to cDNA by RT-PCR. cDNA was PCR amplified using Platinum Taq DNA polymerase (Invitrogen, Breda, the Netherlands) on a Bio-Rad iCycler apparatus (Bio-Rad, USA). Thermal cycling conditions were designed as follows: initial denaturation at 95°C for 10 min, followed by 40 cycles at 95°C for 10 sec and 57°C for 20 sec, and an extension step of 5 min at 72°C. The primer pairs used for PCR were as follows: 5′-GAACAACTACCCATATTTAAAGCTCA-3′ and 5′-GAATCAGGAAGTTCCCTCCA-3′ for mtTFA; 5′-CCAACCGCGAGAAGATGA-3′ and 5′-CCAGAGGCGTACAGGGATAG-3′ for β-actin. Expected amplicon sizes were 95 bp and 97 bp. The specificity of the amplification was verified by melt curve analysis and evaluation of efficiency of PCR amplification. Transcript levels of the constitutive housekeeping gene product β-actin were quantitatively measured for the control of loading. Relative changes in transcript abundance were expressed as ΔC_T_ values (ΔC_T_ = ΔC_T_ reference -ΔC_T_ target), where higherΔC_T_ values indicate higher transcript abundances.

#### Western blot analysis

A total of 40 µg of protein from each sample was separated on 12% SDS polyacrylamide gel, and electrotransferred to a polyvinylidene fluoride membrane. The membrane was blocked with 5% nonfat dry milk in PBS containing 0.05% Tween (PBST) for 1 hour. After washing with PBST, the membrane was incubated with a rabbit monoclonal mtTFA antibody (1∶100; Abcam, USA) overnight at 4°C. The membrane was washed four times and incubated with horseradish peroxidase -conjugated secondary antibody (anti-rabbit, 1∶10,000; Santa Cruz, CA) for 1 hour. The film was developed by enhanced chemiluminescence detection system (ECL, BestBio Pharmacia Biotech). The levels of the constitutive housekeeping gene product β-actin were also measured as control of loading. The protein expression was quantified and expressed in arbitrary units (AU).

#### Immunohistochemistry

Paraffin-embedded sections of human lung tissue were deparaffinized and rehydrated with xylene and ethanol. Antigen retrieval was performed using the microwave method with citrate buffer for 20 minutes. Avidine and biotin block was performed, and endogenous peroxidase was quenched with 3% hydrogen peroxide. After blocking with 5% normal goat serum, a rabbit anti-human mtTFA antibody (1∶100) (Abcam, USA) was applied overnight at 4°C. The sections were washed with PBS with 0.05% Tween and incubated with biotinylated goat antirabbit IgG (1∶10,000; Santa Cruz, CA). After washing, sections were stained with ABC Vectastatin reagents (Wuhan Boster Biological Technology Corporation, Ltd) and DAB (Beijing Zhong Shan-Golden Bridge Biological Technology Corporation). Sections were then scored by a pathologist in a blinded fashion (number of mtTFA-positive endothelial cells/100 endothelial cells per case) for mtTFA staining in capillaries, small/medium arteries.

#### Bisulfite Sequencing PCR (BSP)

Genomic DNA was isolated from the lung tissue using the DNeasy DNA isolation kit (Qiagen, Valencia, CA) followed by EcoRI digestion. Bisulfite modification of genomic DNA was performed using the EZ DNA Methylation-Gold Kit (Zymo Research Corporation, Orange, CA) following the manufacturer's directions. Briefly, 1 µg of purified DNA (20 µl) was incubated in 130 µl of CT Conversion Reagent at 98°C for 10 min and 64°C for 2.5 h, resulting in conversion of unmethylated cytosines into uracil. After DNA purification, the bisulfite-modified DNA was amplified using PCR. The primers were designed using the online program MethPrimer (http://www.urogene.org/methprimer/): PCR primers, 5′-TATTAGAATTTGTTAAATTTTGGGGAAT-3′ and 5′-ACAAACAATCCTACATCCAAAACC-3′. The PCR conditions were as follows: 3 min at 94°C; 35 cycles of 30 sec at 94°C, 30 sec at 56°C, and 40 sec at 72°C; and an extension step of 5 min at 7°C. The PCR products were purified using electrophoresis on 1% agarose gels (QIAquick gel extraction kit; Qiagen), recovered or using ethanol precipitation, and then cloned using ligation into pCR 2.1-TOPO cloning vectors with a TA cloning kit (Takara, USA) followed by using the DH5α competent bacteria (Human Key Laboratory of Medical Epigenomics, the Second Xiangya Hospital, Central-South University). Plasmid DNA isolated from at least five clones for each PCR reaction product were subjected to DNA sequence analysis at the Shanghai Biotechnological Company. As compared with the original genomic mtTFA DNA sequence, the methylation of CpG was determined as appearing unconverted cytosines in the CpG sites in bisulfite-modified DNA.

#### Statistical analysis

Statistical analysis was performed with SPSS version 13.0. Data were presented as Means ± SD. T test compared the difference between groups. Linear correlation analysis was conducted using Pearson product moment correlations. Differences were considered statistically significant if *P*<0.05.

## Results

### Histological analysis of lung tissues

#### The pathological change of lung tissue

**Figure 1:**
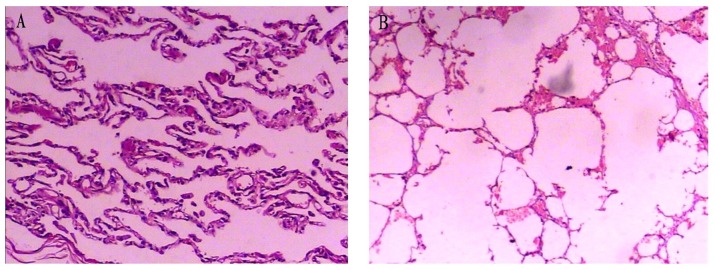
HE staining of the lung tissues. The photomicrographs of lung tissue from the non-COPD group (panel A) and the COPD group (panel B) (magnification: 100×). The mean linear intercept (MLI) and the destructive index (DI) in the COPD group were significantly higher as compared to the non-COPD group (*P<*0.001).

There were significant pathological differences between the non-COPD group and COPD group. The morphology of lung tissue was normal in the non-COPD group. Alveolar septa were intact and there was little inflammatory cell infiltration (Figure 1A). But in the COPD group, there was severe inflammatory infiltration in both airway and alveoli. As shown in Figure 1B, cilium defluxion and goblet cell proliferation were observed in airways, meanwhile destruction and rarefaction were detected in alveoli of COPD patients. Compared to the non-COPD group, the MLI and DI were significantly higher in the COPD group (MLI: 70±23 µm *vs.* 43±6 µm, *P*<0.001; DI: 55±4% *vs.* 16±3%, *P*<0.001).

#### The pathological changes of pulmonary vasculature

**Figure 2:**
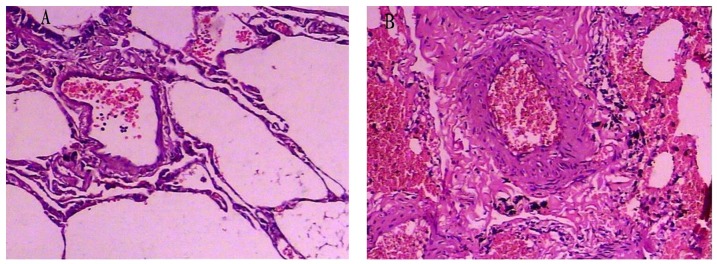
HE staining of the pulmonary vasculature. The photomicrographs of pulmonary vessels from the non-COPD (panel A) and the COPD group (panel B) (magnification: 100×). The histological presentation of pulmonary vasculature between the COPD group and non-COPD group was different.

The morphology of pulmonary vasculature between the non-COPD and COPD groups was different. The pulmonary vasculature was normal and there was little inflammatory cell infiltration in the non-COPD group (Figure 2A). However, in the COPD group, the vessel walls were much thicker, especially in the smooth muscle layer. The lumen was narrowed as a consequence of increased thickness of vascular wall in the COPD group (Figure 2B).

### The pulmonary vascular endothelial apoptosis

**Figure 3:**
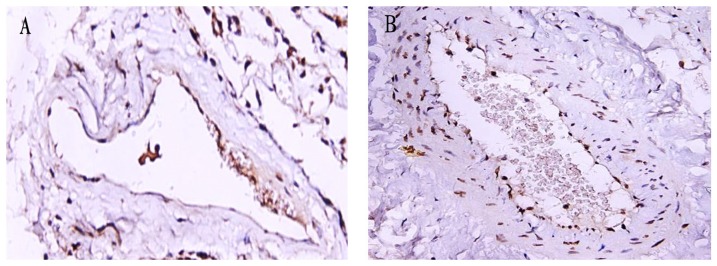
TUNEL assay of middle pulmonary vasculature. The positive cells were stained in yellow brown by TUNEL assay. Photomicrographs of TUNEL stained lung tissue of the non-COPD group (panel A) and the COPD group (panel B) (magnification: 400×). The number of TUNEL positive pulmonary vascular endothelial cells in the COPD group was much higher than that in the non-COPD group.

The apoptosis of pulmonary vascular endothelial cells in the non-COPD and COPD groups was analyzed by TUNEL assay. The apoptotic positive cells were stained in yellow brown in TUNEL assay. The apoptotic index of pulmonary vascular endothelial cells in the COPD group was much higher than that in the non-COPD group (*P*<0.001) (Figure 3 and Table 2).

**Table 2:** The apoptotic index of pulmonary vascular endothelial in non-COPD group and COPD group(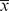
±s).

group	number	Apoptotic index(%)
non-COPD group	10	5.9±1.0
COPD group	11	13.8±1.9***

*** *P*<0.001 compare with non-COPD group.

### Expression of mtTFA mRNA and protein in lung tissue from non-COPD and COPD patients

#### Lung tissue *mtTFA* mRNA detected by RT-PCR and qRT-PCR

**Figure 4:**
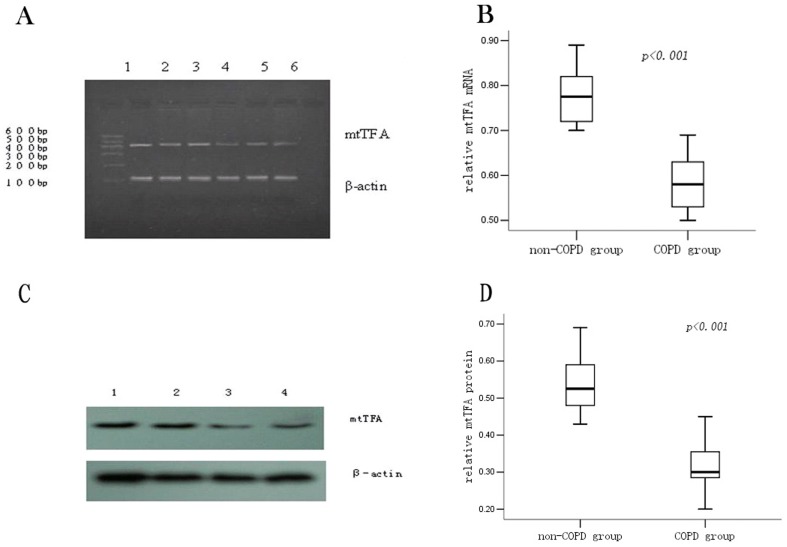
Expression of the mtTFA mRNA and protein in the lung tissue. Expression of the pulmonary *mtTFA* mRNA measured by RT-PCR (panel A). Line 1, 2 and 3 were the non-COPD patients and line 4, 5 and 6 were the COPD patients. The level of mtTFA mRNA in lung tissues from the COPD group was lower as compared to the non-COPD group. Expression of the pulmonary *mtTFA* mRNA measured by qRT-PCR (panel B). Box plots display the range and distribution of the data with the upper, lower quartiles, and the median of the maximum difference of *mtTFA* mRNA in the non-COPD group and the COPD group. The median for each dataset is also indicated by the centerline. Expression of mtTFA protein (panel C): Line 1 and 2 were the non-COPD patients and line 4 and 5 were the COPD patients. The quantitative analysis of the protein density is also shown (panel D).

We analyzed the expression of *mtTFA* mRNA in lung tissues by semi-quantitative RT-PCR. Agarose gel electrophoresis showed expression of *mtTFA* mRNA in lung tissue from the COPD patients was lower as compared to that from the non-COPD patients (Figure 4A). Furthermore, expression of *mtTFA* mRNA in the lung tissue was also determined by qRT-PCR. The relative amount of *mtTFA* mRNA expression in the COPD patients was lower than that in the non-COPD patients (COPD group: 0.59±0.07 *vs.* non COPD group: 0.78±0.09, *P*<0.001) (Figure 4B).

#### Expression of the mtTFA protein of lung tissue in non-COPD and COPD patients

**Figure 5:**
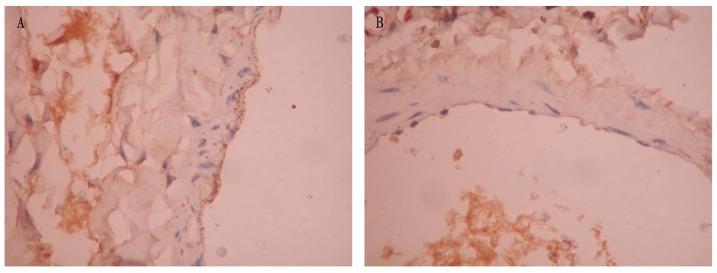
Expression of mtTFA protein in the lung of the non-COPD and COPD groups by immunohistochemistry. mtTFA protein expression of the non-COPD group (panel A) and the COPD group (panel B) (magnification: 400×). mtTFA protein was yellow brown in DAB staining. When compared to the non-COPD group, the expression of mtTFA protein was lower in the COPD group.

We next measured expression of the mtTFA protein in lung tissue by Western blot. The relative mtTFA protein expression in the COPD group was lower than that in the non-COPD group (COPD: 0.32±0.07 *vs.* non-COPD: 0.55±0.09, *P*<0.001) (Figure 4C & 4D). To further characterize the localization of pulmonary mtTFA protein, immunohistochemical staining on lung tissue sections using an antibody specific for mtTFA was performed. mtTFA protein was mainly localized in endothelial cells of blood vessels, and consistent with Western blot result, its expression was lower in the COPD group as compared with the non-COPD group (Figure 5).

### The methylation status of *mtTFA* promoter in lung tissue of non-COPD *vs*. COPD patients

**Figure 6:**
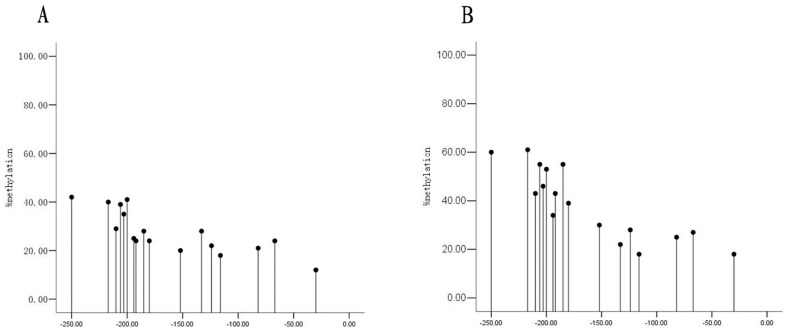
The methylation pattern of mtTFA in the non-COPD and the COPD groups. The model of mtTFA methylation of the non-COPD group (panel A) and the COPD group (panel B).

**Figure 7:**
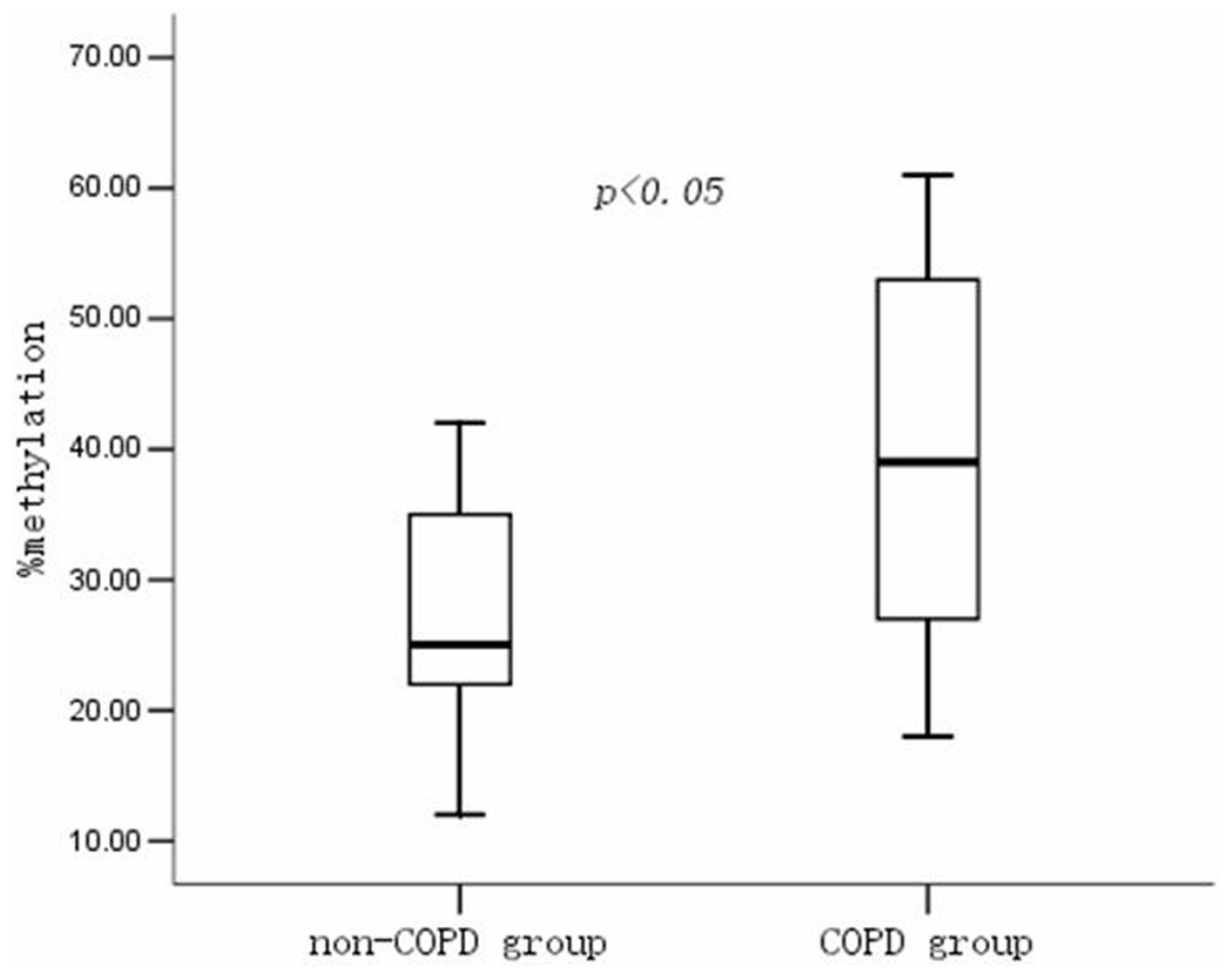
The percentage of *mtTFA* promoter methylation in the non-COPD and COPD groups. Box plots display the extremes, the upper and lower quartiles, and the median of the maximum difference in the non-COPD and COPD groups. The median for each dataset is indicated by the centerline, *P* value of methylation percentage of *mtTFA* promoter was 0.013 between the non-COPD and the COPD group.

We further determined the status of *mtTFA* promoter methylation in the lung tissue from the non-COPD and COPD patient by BSP and DNA sequence analysis. The *mtTFA* promoter methylation pattern of lung tissues from non-COPD and COPD patients are shown in Figure 6A and 6B. The percentage of *mtTFA* promoter methylation (%) in COPD patients was higher as compared with non-COPD patients (COPD: 38.6±14.7 *vs.* non-COPD 27.8±8.8; *P*<0.05) (Figure 7).

### Correlation analysis

#### The correlation analysis of endothelial cell apoptosis with lung function and smoking index

**Figure 8:**
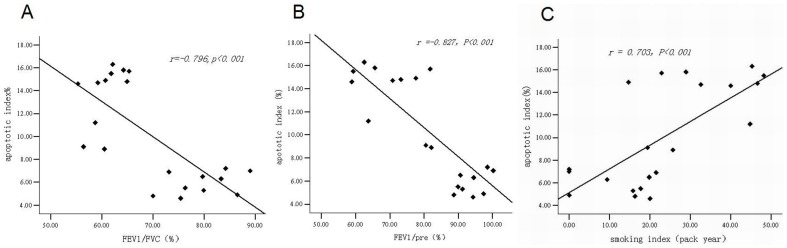
The scatterplot of correlation analysis of vascular endothelial apoptotic index and FEV_1_/FVC, FEV_1_/Pre, smoking index. The scatterplot of correlation analysis of endothelial cell apoptotic index and FEV_1_/FVC (*r* = −0.796, *P*<0.001) (panel A). The scatterplot of correlation analysis of endothelial cell apoptotic index and FEV_1_/Pre (*r* = −0.827, *P*<0.001) (panel B). The scatterplot of correlation analysis of endothelial cell apoptotic index and smoking index (*r* = 0.703, *P*<0.001) (panel C).

The endothelial cell apoptotic index was negatively correlated with FEV_1_/FVC (*r* = −0.796, *P<*0.001) and FEV_1_/Pre (*r* = −0.827, *P<*0.001) (Figure 8A & 8B) and positively correlated with smoking index (*r* = 0.703, *P<*0.001) (Figure 8C).

#### The correlation analysis of lung tissue mtTFA protein and FEV_1_/Pre, smoking index, endothelial cell apoptotic index

**Figure 9:**
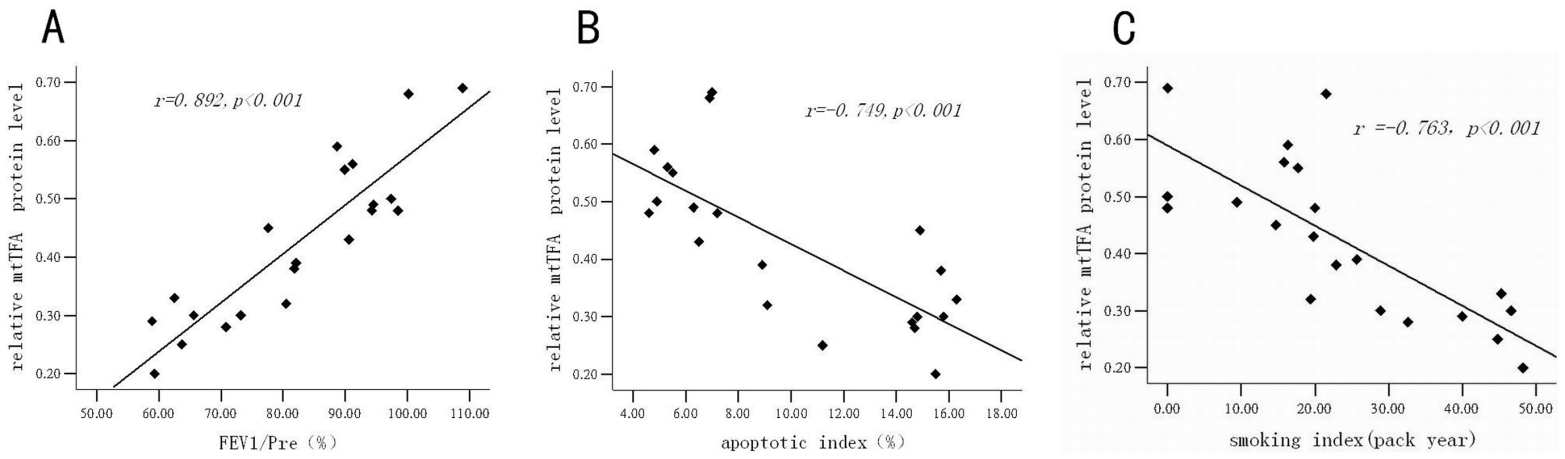
The scatterplot of correlation analysis of lung tissue mtTFA protein and FEV_1_/Pre, vascular endothelial apoptotic index, smoking index. The scatterplot of correlation analysis of lung tissue mtTFA protein and FEV_1_/Pre (*r* = 0.892, *P*<0.001) (panel A). The scatterplot of correlation analysis of lung tissue mtTFA protein and endothelial apoptotic index (*r* = −0.749, *P*<0.001) (panel B). The scatterplot of correlation analysis of lung tissue mtTFA protein and smoking index (*r* = −0.763, *P*<0.001) (panel C).

Expression of mtTFA protein of lung tissue was positively correlated with FEV_1_/Pre (*r* = 0.892, *P<*0.001) (Figure 9A), negatively correlated with vascular endothelial cell apoptotic index (*r* = −0.749, *P<*0.001) and smoking index (*r* = −0.763, *P<*0.001) (Figure 9B & 9C).

## Discussion

In this study we found that the vascular endothelial cell apoptotic index in COPD patients was much higher as compared with non-COPD patients, and it was negatively correlated with FEV_1_/Pre, but positively correlated with smoking index. We demonstrated that 1) the expression of mtTFA mRNA and protein was lower in the lung tissue of COPD patients with squamous lung cancer as compared to squamous lung cancer patients without COPD; 2) the expression of mTFA was positively correlated with vascular endothelial cell apoptotic index; 3) but was negatively correlated with cigarette smoking index. We also found that the percentage of *mtTFA* promoter methylation was higher in the COPD group compared to non-COPD group.

COPD is a leading cause for chronic morbidity and mortality worldwide, and it has caused a substantial economic and social burden [23]. Several mechanisms are involved in the pathogenesis of disease. They include 1) inflammatory infiltration; 2) imbalance between proteolytic and anti-proteolytic activity; 3) oxidative stress; and 4) apoptosis/proliferation imbalance [3]. One of the consequences following these processes is cellular apoptosis, a complex and well-regulated process that leads to cell death. Presence of higher apoptotic level in the human emphysematous lung has been reported [24], [25]. It has been proposed that epithelial and endothelial cell death due to a decrease in cell maintenance factors may be responsible for the development of emphysema. Consistent with this notion, COPD can be re-produced by inducing pulmonary endothelial apoptosis in animal models [26], [27]. Similar to these observations, we found an increased number of apoptotic pulmonary endothelial cells in the COPD patients as compared to the non-COPD patients. The apoptotic index was positively correlated with FEV_1_/Pre, FEV_1_/FVC and smoking index [6], [28]. These results indicate that pulmonary endothelial apoptosis may play an important role in the pathogenesis of COPD.

mtTFA is an important factor regulating mitochondrial biogenesis. It activates transcription and replication of mtDNA [29]. It plays a very important role in the regulation of mitochondrial respiration, anti-oxidative stress and cell apoptosis. Previous study by Remels et al [11] suggested that mtTFA might be involved in the pathogenesis of COPD. We found that the expression of mtTFA mRNA and protein was decreased in the lung tissues from COPD patients as compared to non-COPD patients. The expression of mtTFA was positively correlated with FEV_1_/Pre and FEV_1_/FVC, and negatively correlated with smoking index and vascular endothelial cell apoptotic index. Consistent with previous observations, our results also suggest a role of mtTFA in the apoptosis of pulmonary endothelial cells and in the pathogenesis of COPD. However, the underlying mechanisms responsible for this are poorly understood. There is accumulating evidence that epigenetics may play an important role in the pathogenesis of some respiratory diseases such as asthma and COPD [13]. Interestingly, we found that the percentage of *mtTFA* promoter methylation in lung tissue of the squamous cell lung cancer patients with COPD is much higher as compared to squamous cell lung cancer patients without COPD, suggesting epigenetic regulation may be responsible for the expression of mtTFA.

COPD and lung cancer are both leading causes of morbidity and mortality worldwide. They share a common environmental risk in cigarette smoke exposure and a genetic predisposition. The risk of lung cancer in patients with COPD has been suggested for over 30 years [30], and although tobacco exposure remains as an important confounding variable, an independent association likely exists. Punturieri et al [31] suggested that lung cancer and COPD might be two sides of the same coin. Because DNA methylation, histone deacetylation, and protein phosphorylation are involved in the pathogenesis of lung cancer [32]–[34], it is suggested that epigenetic modifications may also attribute to the pathogenesis of COPD, either alone or when associated with lung cancer [35], [36]. Demeo et al [14] found that DNA methylation status at distinct CpG loci was associated with both the presence and severity of COPD and lung function, suggesting that DNA methylation may be a biomarker of COPD and may highlight new pathways of COPD pathogenesis.

Aberrant methylation of the promoter region of oncogenes and tumor suppressor genes plays an important role in the pathogenesis of lung cancer and the methylation status is closely related to smoking [37], [38]. There are many carcinogens in tobacco smoke. In addition, tobacco smoke is a mucosal irritant that induces inflammation, resulting in the generation of oxygen-free radicals. Furthermore, smoking increases the activity of DNA methyltransferase [39] which drives the *de novo* hypermethylation of susceptible loci [40]. These direct or indirect interactions could be involved in the enhanced DNA methylation in heavy smokers. Two recent studies reported molecular alterations in spontaneous sputum of cancer-free heavy smokers [41], [42]. Athough there are different opinions about the effect of smoke on DNA methylation [43]–[45]. There is no doubt that smoke can affect methylation of either global or individual genes.

CpG islands in gene promoters represent a major target for DNA hypermethylation which contributes to gene silencing by inhibiting the binding of certain transcription factors to their recognition sequences, attracting methylated DNA-binding proteins, and/or chromatin remodeling. The mtTFA promoter contains a large CpG island that spans the proximal promoter and exon, encompassing the start site for transcription. Genomic DNA methylation can result in silencing of *mtTFA* and would be one of the possible mechanisms responsible for mitochondrial biogenesis and respiration [17]. These findings suggest that *mtTFA* methylation may be implicated in the development and progress of COPD associated with cigarette smoke. In our study, the sample size was small and the patients with COPD had fairly mild disease as most lung cancer patients who undergo surgical resection have relatively preserved lung function. We plan to enroll more patients with different stages of COPD and an additional group of COPD patients with no smoking history. In spite of this, our findings regarding alteration of *mtTFA* methylation in COPD might provide important information for future investigations of COPD.
